# Changing prevalence of chronic hepatitis B virus infection in China between 1973 and 2021: a systematic literature review and meta-analysis of 3740 studies and 231 million people

**DOI:** 10.1136/gutjnl-2023-330691

**Published:** 2023-10-05

**Authors:** Zhenqiu Liu, Chunqing Lin, Xianhua Mao, Chengnan Guo, Chen Suo, Dongliang Zhu, Wei Jiang, Yi Li, Jiahui Fan, Ci Song, Tiejun Zhang, Li Jin, Catherine De Martel, Gary M Clifford, Xingdong Chen

**Affiliations:** 1 State Key Laboratory of Genetic Engineering, Human Phenome Institute, and School of Life Sciences, Fudan University, Shanghai, China, Shanghai, China; 2 Fudan University Taizhou Institute of Health Sciences, Taizhou, China; 3 National Clinical Research Center for Cancer, National Cancer Center, Cancer Hospital, Chinese Academy of Medical Sciences and Peking Union Medical College, Beijing, China; 4 Department of Medicine, Li Ka Shing Faculty of Medicine, University of Hong Kong, Hong Kong, Hong Kong Special Administrative Region, China; 5 Department of Epidemiology, School of Public Health, Fudan University, Shanghai, China; 6 Key Laboratory of Public Health Safety, Fudan University, Ministry of Education, Shanghai, China; 7 Department of Epidemiology, School of Public Health, Nanjing Medical University, Nanjing, China; 8 Early Detection, Prevention and Infections Branch, International Agency for Research on Cancer (IARC/WHO), Lyon, France; 9 National Clinical Research Center for Aging and Medicine, Huashan Hospital, Fudan University, Shanghai, China; 10 Yiwu Research Institute of Fudan University, Yiwu, China

**Keywords:** META-ANALYSIS, HEPATITIS B

## Abstract

**Objective:**

China concentrates a large part of the global burden of HBV infection, playing a pivotal role in achieving the WHO 2030 global hepatitis elimination target.

**Methods:**

We searched for studies reporting HBV surface antigen (HBsAg) seroprevalence in five databases until January 2023. Eligible data were pooled using a generalised linear mixed model with random effects to obtain summary HBsAg seroprevalence. Linear regression was used to estimate annual percentage change (APC) and HBsAg prevalence in 2021.

**Results:**

3740 studies, including 231 million subjects, were meta-analysed. HBsAg seroprevalence for the general population decreased from 9.6% (95% CI 8.4 to 10.9%) in 1973–1984 to 3.0% (95% CI 2.1 to 3.9%) in 2021 (APC=−3.77; p<0.0001). Decreases were more pronounced in children <5 years (APC=−7.72; p<0.0001) and 5–18 years (−7.58; p<0.0001), than in people aged 19–59 years (−2.44; p<0.0001), whereas HBsAg seroprevalence increased in persons ≥60 years (2.84; p=0.0007). Significant decreases were observed in all six major Chinese regions, in both men (APC=−3.90; p<0.0001) and women (−1.82; p<0.0001) and in high-risk populations. An estimated 43.3 million (95% uncertainty interval 30.7–55.9) persons remained infected with HBV in China in 2021 (3.0%), with notable heterogeneity by region (<1.5% in North China to>6% in Taiwan and Hong Kong) and age (0.3%, 1.0%, 4.7% and 5.6% for <5 years, 5–18 years, 19–59 years and ≥60 years, respectively).

**Conclusions:**

China has experienced remarkable decreases in HBV infection over the last four decades, but variations in HBsAg prevalence persist in subpopulations. Ongoing prevention of HBV transmission is needed to meet HBV elimination targets by 2030.

**Trial registration number:**

PROSPERO (CRD42021284217)

**WHAT IS ALREADY KNOWN ON THIS TOPIC**

- Chronic HBV infection remains one of the leading causes of liver-related morbidity and mortality.
- China carries the heaviest burden of HBV infections worldwide, holding a crucial position in meeting the WHO’s goal of global hepatitis eradication by 2030.

**WHAT THIS STUDY ADDS**

- We conducted a broad review and meta-analysis on HBV infection in China’s population using 3740 publications.
- There has been a significant decline in national HBV surface antigen prevalence from 9.6% in 1973–1984 to 3.0% in 2021, with varying degrees of decrease across demographics.
- An estimated 43.3 million persons remained infected with HBV in China in 2021, with notable heterogeneity by region.

**HOW THIS STUDY MIGHT AFFECT RESEARCH, PRACTICE OR POLICY**

- This study provides the most comprehensive and robust evidence to date of China’s progress, by geographical region and other strata of the population, towards meeting the goal of HBV elimination by 2030.
- Our findings demonstrate the impact of HBV vaccination over the last three decades and highlight a continuous need for ongoing prevention of HBV transmission and expanded HBV treatment.

## Introduction

Chronic HBV infection remains a global public health concern, causing considerable liver-related morbidity and mortality.^1–3^ In 2015, the UN Sustainable Development Goals (SDGs) proposed an SDG-2030 target of ‘combating hepatitis’, and the WHO launched a strategy to eliminate hepatitis as a public health threat by 2030.^4^ Given that a large fraction of the global HBV-attributable disease burden concentrates in China alone,^5^ combating HBV infection in China is pivotal to these strategies.^6^

HBV has long been known to be endemic in China and a large nationwide sero-survey conducted in 1992 reported a 9.8% prevalence of chronic HBV infection in the general population, as measured by HBV surface antigen (HBsAg).^7^ To combat the HBV epidemic, the Chinese government subsequently implemented a series of programmes to reduce HBV transmission, including incorporation of HBV vaccination into the routine immunisation schedule for infants in 1992 and abolishment of patient copayments for vaccines in 2005.^8^ These national efforts are known to have reduced chronic HBV infection in China, particularly in infants and children,^9^ as shown in repeat nationwide surveys in 2006 and 2014. Nevertheless, the prevalence of chronic HBV infection, and its decrease over time, is expected to vary by geographical region and across other strata of the Chinese population.^10^

A detailed vision of the current epidemiology of chronic HBV infection is key to the ongoing provision of HBV prevention efforts such as strategies for HBV screening and treatment,^11^ but is challenging across the vast population of China. While some routine medical surveillance systems do exist in China, they cannot capture asymptomatic infections, and are incomplete in their coverage.^10 12^ Furthermore, although many seroepidemiological surveys in the general population are publicly available, either in scientific journals or governmental reports, data are scattered over region and over time.

Thus, we aimed to provide a comprehensive overview of the changing seroepidemiology of HBV infection in China. To this end, we undertook a systematic literature review and meta-analysis of data on HBsAg seroprevalence, and produced pooled estimates by period, geography and other subgroups of the general population, as well as in groups known to be at high risk for HBV infection. Our pooled estimates include, and build on, previous nationwide surveys,^9 13^ to define the public health efforts still needed to achieve HBV elimination in China.

## Methods

### Search strategy and study selection

The current study was registered in PROSPERO (CRD42021284217). We conducted a literature search in English databases, Medline, EmBase, and Web of Science, and in Chinese databases, WanFang and China National Knowledge Infrastructure, up to 1 July 2021, and updated our search until 31 January 2023. Detailed search strategies are shown in the online supplemental material. In total, 18 285 publications were identified and catalogued using Endnote V.X9 (Clarivate Analytics, Boston, Massachusetts, USA). The publication screening process is shown in the online supplemental figure S1. Two groups of authors (ZL and XM; WJ and DZ) independently screened the titles and abstracts of all articles identified, according to predefined inclusion and exclusion criteria, similar to those used previously.^14^ Any disagreement was resolved by discussion with another author (CL). To be eligible for inclusion, a study should (1) Report results of a serological test for HBsAg; (2) Provide data allowing estimation of HBsAg prevalence; and (3) Include Chinese people living in mainland China, Macau, Hong Kong, and Taiwan as study subjects. Exclusion criteria were: (1) Full text not available; (2) Publications in languages other than Chinese or English; (3) Studies conducted in duplicated population; (4) HBV diagnosis based on non-serum test or using finger rapid test; (5) Studies among people who suffered from liver diseases; (6) Meta-analysis, surveillance registration or national notifiable disease reports of incident hepatitis virus cases; and (7) Case studies. A total of 3740 eligible studies were selected for analyses (online supplemental figures S1–S3).

10.1136/gutjnl-2023-330691.supp1 Supplementary data

### Data collection and processing

Two groups of authors (ZL, XM and CG; CL, C.Suo and WJ) independently extracted data from eligible studies, with any inconsistencies resolved by discussion. Number of persons tested for HBsAg and number of HBsAg-positive persons were extracted for each study, each of which were additionally classified as being derived from general or high-risk population samples (full definition described in the online supplemental material). High-risk populations were categorised as people living with HIV (PLWH), injecting drug users (IDU), sex workers, men who have sex with men (MSM), prisoners, migrant workers, or hospital patients (both inpatients and outpatients, but excluding those diagnosed with liver diseases). For general population studies, as far as possible, we further categorised populations by: (1) Province and/or region, (2) Rural versus urban, (3) Age (<5 years, 5–18 years, 19–59 years and ≥60 years) (full definitions described online supplemental material), (4) Sex (male/female), (5) Type of HBV assay (ELISA vs other), (6) Publication language (Chinese vs English), (7) Study sample size (<500, 500–1000, 1001–10 000, and >10 000), and (8) Study grade. Publications classified as grade A were characterised as having (1) Multisite participants recruited with stratified multistage cluster sampling, (2) No restriction on age and sex of participants, and (3) Sample size >1000. Studies conducted in a single site with participants recruited by random sampling were classified as grade B. Studies conducted in a single site and for which the participants were not randomly recruited (eg, check-up for new military recruits) and those that did not report year of sample collection were classified as grade C.

In order to assess temporal trends, each study was categorised according to year of sample collection. Studies which reported sample collection over 2–5 years were attributed median calendar year. Mean lag time between sample collection and paper publication was 2.5 years. Thus, for studies not reporting a specific sample collection period (1.7% of total publications, 0.01% of subjects), we attributed the year of publication minus 3 as a surrogate for year of sample collection. Studies that reported collection across >5 years, and that did not report annual results (1.3% of total publications, 0.005% of subjects), were excluded from temporal analyses. Study years were additionally grouped into 2-year periods, and again into three intervals based on important programmatic milestones (see above),^8^ namely 1973–1992, 1993–2005 and 2006–2021.

### Statistical analysis

Pooled estimates of HBsAg seroprevalence were calculated by meta-analysis using a generalised linear mixed model with random effects.^15^ Corresponding 95% CIs were calculated with the exact method based on binomial distribution. Based on 2 yearly nationwide estimates, a segmented linear regression model^16^ identified no statistically significant breakpoint in the trend of HBsAg seroprevalence over time. Thus, temporal trends were estimated by a regression line that fitted the natural logarithm of the HBsAg prevalence, that is, *ln(y) = α + βx + ɛ,* where *y=*crude prevalence in each study and *x*=study calendar year. In this trend analysis, due to small sample sizes, HBsAg seroprevalence data in 1973–1984 were integrated into a single estimate and set to represent a starting point in 1984. We used annual percentage change (APC) to quantify the overall temporal trends of HBsAg prevalence from 1984 to 2021, calculated as 100×(*exp*(*β*)−1).^17^ In order to overcome the underlying impact of small sample sizes on APC estimates, the logarithmic transformed sample size was set as the weight in the linear regression model. We additionally fitted a linear regression model (ie, *y = α + βx + ɛ,* where *y=*crude prevalence in each study and *x*=study calendar year) to present the absolute change in HBsAg prevalence that was expressed as the regression slope.

Using this linear regression model, we estimated the HBsAg prevalence in 2021 nationwide based on data in 1973–2020, for six regions in mainland China, Taiwan and Hong Kong. Log-transformed population size of each study was set as the regression weight. We also calculated the national seroprevalence and number of HBV carriers by weighing by corresponding population size in 2021, that is, $N=\sum Pre_{i}\times Pop_{i}$ , where ${Pre}_{i}$ and ${Pop}_{i}$ denotes the prevalence and population size in each region/territory, respectively. The prediction model was also applied for subpopulations by sex and age. Of note, for prediction of HBsAg seroprevalence in people aged under 5 years and 5–18 years, we first log-transformed the prevalence data to avert a prediction<0. The predicted values were then exponentially transformed to represent the HBsAg prevalence in these populations.

Meta-regression was performed to further examine potential contributors to between-study heterogeneity, expressed as prevalence ratios using meta-regression models incorporating year of study (1973–1992, 1993–2005 and 2006–2021), HBV testing assay, study grade, sample size, publication language, geographical region and/or population category (general population vs high-risk groups). Age group (under 5 years, 5–18 years, 19–59 years and ≥60 years) and sex were evaluated in a meta-regression model restricted only to studies providing age-specific or sex-specific HBsAg seroprevalence, respectively.

Two sensitivity analyses were performed to calculate the national and regional seroprevalence of HBsAg, first using only publications with the ‘Grade A’ from each province, and second excluding healthcare workers and blood donors. The percentage of total variation due to between-study heterogeneity was evaluated using the *I*² measure. Publication bias was evaluated by the Egger test.

Segmented linear regression was performed using the *segmented* package (1.5–0) and meta-analyses were implemented using the *meta* (4.18–0) and *metafor* (2.4–0) packages in R program (V.4.1.1). We applied false discovery rate (FDR) to correct the p values of multiple testing. An FDR <0.05 was deemed statistically significant.

## Results

### Trends in HBsAg seroprevalence in the general population

**Figure 1:**
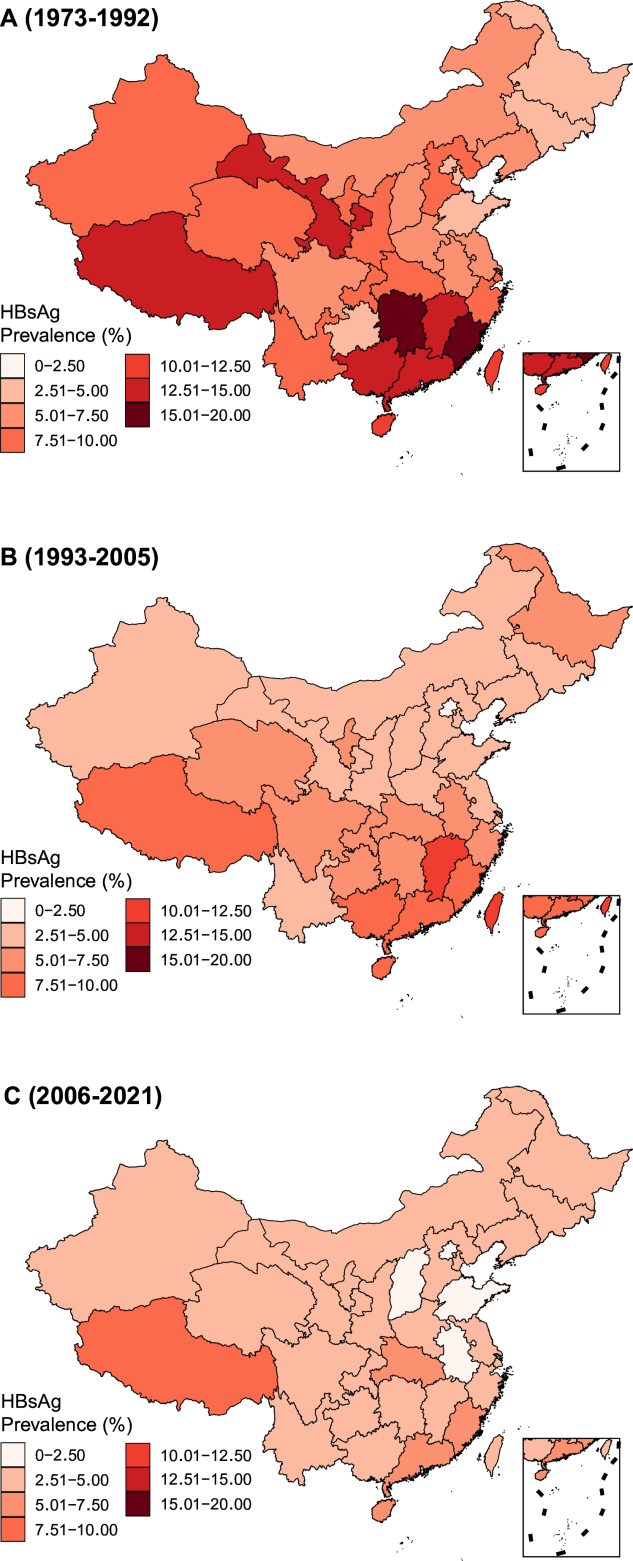
The seroprevalence of hepatitis B surface antigen (HBsAg) at the provincial level by study period.

A total of 3740 publications involving more than 231 million subjects were included. Eligible studies covered all six regions (and all 31 provinces) in mainland China, as well as Hong Kong and Taiwan (online supplemental figure S2). For the general population, 3283 studies reported HBsAg prevalence, including 224 million subjects (table 1). Nationwide, the pooled HBsAg seroprevalence decreased from 8.2% (95% CI 7.5 to 9.0%) in 1973–1992 to 5.7% (95% CI 5.5 to 6.0%) in 1993–2005 and 3.6% (95% CI 3.5 to 3.8%) in 2006–2021 (table 1). HBsAg seroprevalence varied across the main regions/territories of China, all of which observed decreases in HBsAg seroprevalence across the three periods, with the notable exception of Hong Kong (table 1). Declines in HBsAg seroprevalence across the three periods were also observed at the provincial level (figure 1; online supplemental table S1). Tibet was the only province where HBsAg seroprevalence increased between 1993–2005 (8.2%, 95% CI 5.3 to 12.5%) and 2006–2021 (8.8%, 95% CI 6.4 to 12.1%) (online supplemental table S1). Trends in HBsAg seroprevalence across the three periods are also shown stratified by strata of sex and age, as well as for rural versus urban areas (online supplemental table S2).

**Table 1:** HBsAg seroprevalence in the general population in China, 1973–2021

Region	No. of study*	Sample size	HBsAg seroprevalence (%, 95% CI) by study period†					
			1973–1992		1993–2005		2006–2021	
			Sample size	HBsAg seroprevalence (%, 95% CI)	Sample size	HBsAg seroprevalence (%, 95% CI)	Sample size	HBsAg seroprevalence (%, 95% CI)
China mainland								
South Central China	1081	27 034 934	192 511	11.0 (9.7 to 12.6)	14 412 661	7.3 (6.9 to 7.8)	12 429 762	4.5 (4.1 to 4.9)
East China	1003	24 286 329	165 400	7.6 (6.3 to 9.2)	4 954 938	5.2 (4.9 to 5.6)	19 165 991	3.3 (2.9 to 3.6)
South-West China	330	14 253 995	85 302	7.6 (5.8 to 9.8)	1 020 232	6.1 (5.4 to 6.8)	13 148 461	3.8 (3.3 to 4.2)
North China	306	8 275 762	26 341	6.0 (4.5 to 7.9)	988 880	3.1 (2.7 to 3.5)	7 260 541	2.5 (2.2 to 2.8)
North-West China	269	3 263 546	9059	10.0 (8.1 to 12.3)	1 195 040	5.1 (4.5 to 5.7)	2 059 447	3.5 (3.0 to 4.0)
North-East China	200	2 761 334	158 813	4.4 (3.2 to 5.9)	1 661 369	4.2 (3.6 to 4.9)	941 152	3.1 (2.7 to 3.6)
Taiwan	50	789 153	26 957	12.3 (9.8 to 15.2)	497 590	10.8 (8.7 to 13.4)	264 606	5.0 (3.0 to 8.2)
Hong Kong	13	399 145	3569	3.6 (3.0 to 4.3)	226 326	8.8 (7.4 to 10.4)	169 250	8.8 (7.9 to 9.7)
Nationwide‡	3283	224 064 312	742 940	8.2 (7.5 to 9.0)	29 461 833	5.7 (5.5 to 6.0)	193 859 539	3.6 (3.5 to 3.8)

*The total study number was not exactly equal to the sum of study number in each region because there were multiple regions involved in certain studies.

†*I^2^* for interstudy heterogeneity >90% (p<0.0001) for all strata in this table, except for the period 1973–1992 of Hong Kong.

‡Data from studies without specific information on province were also included.

HBsAg, HBV surface antigen.

**Figure 2:**
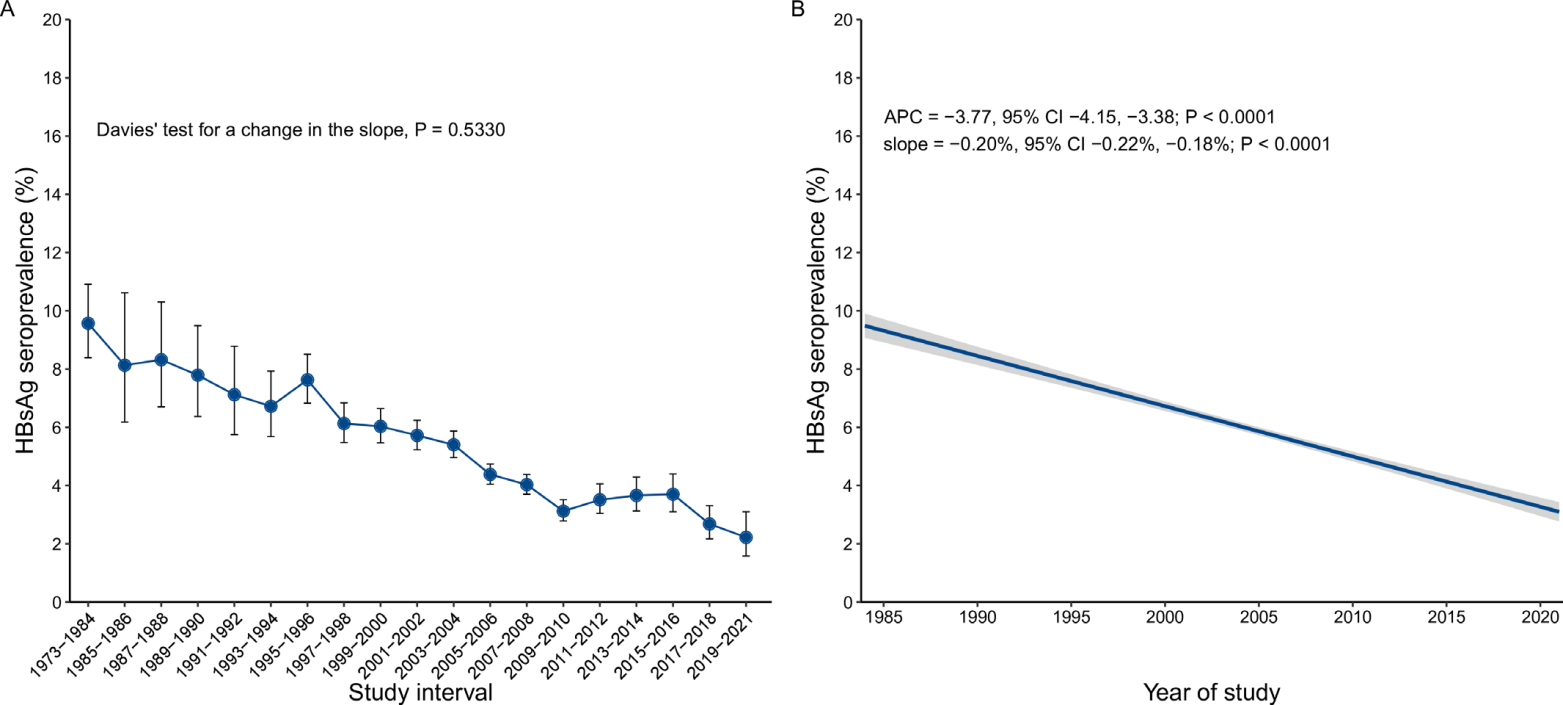
Temporal trends of hepatitis B surface antigen (HBsAg) seroprevalence among the general population in China between 1973 and 2021. (A) Pooled estimates of HBsAg seroprevalence by 2-year study intervals. Studies conducted before year 1984 were compiled due to small sample size and data sparsity. The error bar denotes the 95% CI. (B) Average annual percentage change (APC) of HBsAg seroprevalence. The blue line denotes the fitted values of the linear regression model, in which HBsAg prevalence was regressed on year of study and the log-transformed sample size was used as the weight. The grey shadows denoted the 95% CI of the fitted values. The points denoting prevalence estimate of each included study were not shown due to high overlaps.

Analyses by finer time periods suggested a constant decline in HBsAg seroprevalence nationwide between 1973–1984 and 2019–2021 (figure 2A), which was highly significant in a linear regression model (−0.20% per year, p<0.0001; APC=−3.77, 95% CI −4.15 to –3.38) (figure 2B). APC models showed significant decreases in HBsAg seroprevalence in all six Chinese regions (online supplemental figure S4) and most individual provinces/areas (online supplemental figure S5), with the notable exceptions of Hong Kong (APC=−0.11, 95% CI −1.68 to 1.63).

**Figure 3:**
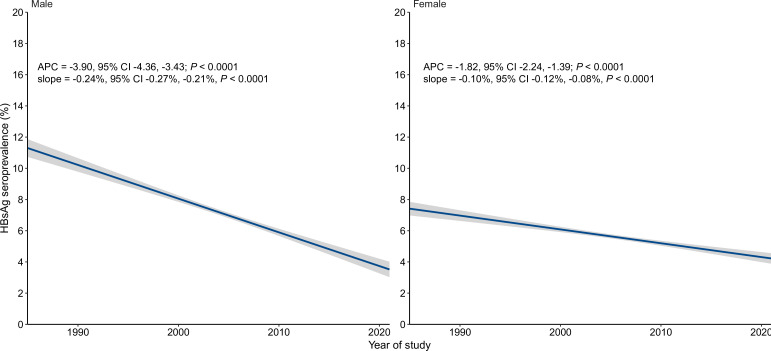
The temporal trend of hepatitis B surface antigen (HBsAg) seroprevalence among the general population in China by gender. The blue lines denote the fitted values of the linear regression model, in which HBsAg prevalence was regressed on year of study and the log-transformed sample size was used as the weight. The grey shadows denoted the 95% CIs of the fitted values. The points denoting prevalence estimate of each included study were not shown due to high overlaps. APC, annual percentage change.

A total of 1608 and 1802 eligible studies, including 16.5 and 120.7 million subjects, respectively, contributed to nationwide meta-analyses (online supplemental table S2) and APC analyses (figure 3) for men and women. HBsAg seroprevalence declined significantly in both men (from 8.8% in 1973–1992 to 4.6% in 2006–2021) and women (from 6.3% in 1973–1992 to 3.9% in 2006–2021), but this decline was greater in men (APC=−3.90, 95% CI −4.36 to –3.43) than women (APC=−1.82, 95% CI −2.24 to –1.39) (figure 3).

**Figure 4:**
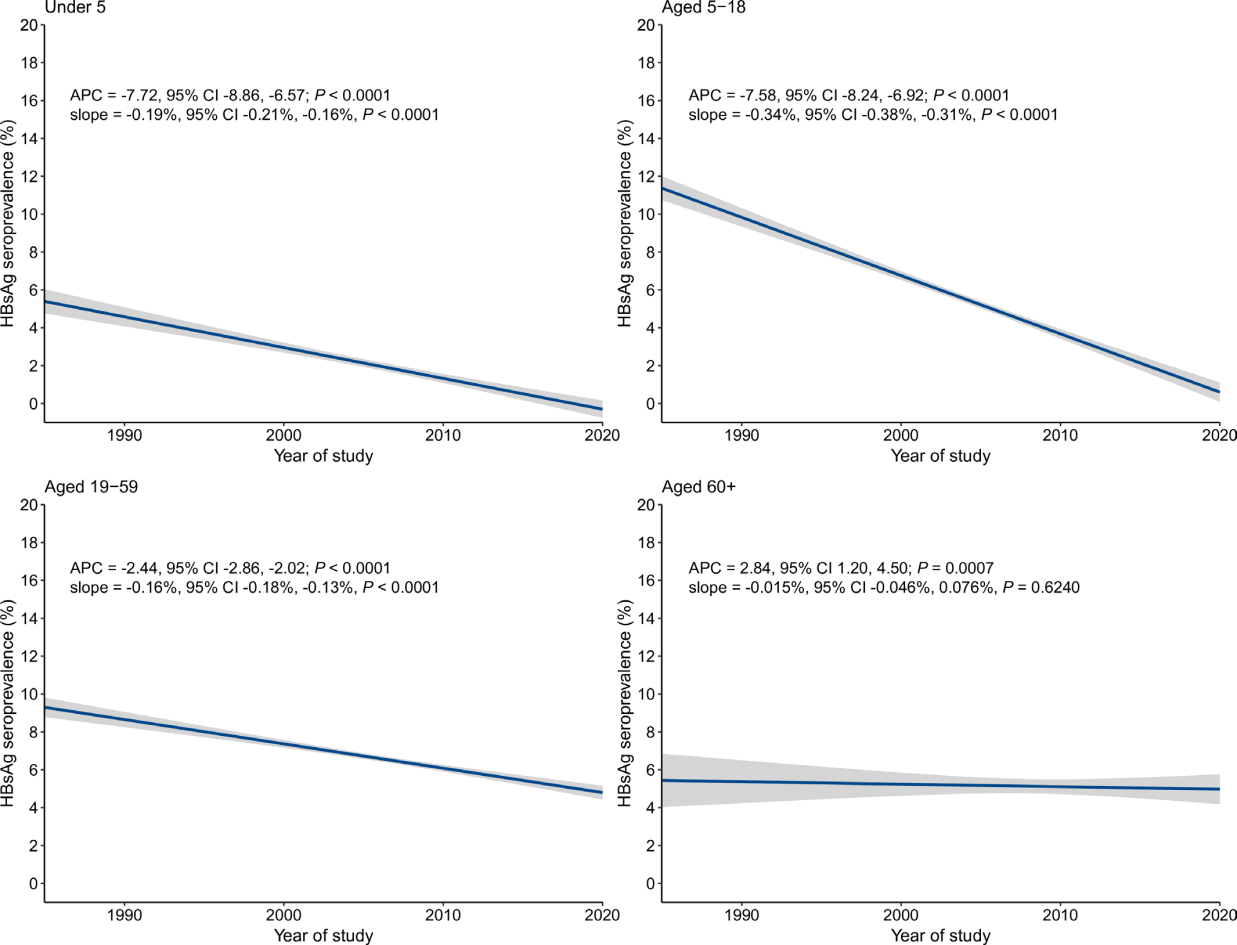
The temporal trend of hepatitis B surface antigen (HBsAg) seroprevalence among the general population in China by age. The blue lines denote the fitted values of the linear regression model, in which HBsAg prevalence was regressed on year of study and the log-transformed sample size was used as the weight. The grey shadows denoted the 95% CIs of the fitted values. The points denoting prevalence estimate of each included study were not shown due to high overlaps.APC, annual percentage change.

Variation in HBsAg seroprevalence trends by age are shown according to nationwide meta-analyses (online supplemental table S2) and APC analyses (figure 4). Strongest declines were observed in children aged <5 years (409 studies, nearly one million subjects; from 5.0% in 1973–1992 to 0.6% in 2006–2021, APC=−7.72, 95% CI −8.86 to –6.57) and 5–18 years (910 studies, 5.1 million subjects; from 10.1% in 1973–1992 to 2.4% in 2006–2021, APC=−7.58, 95% CI −8.24 to –6.92) (online supplemental table S2, figure 4). A more moderate, although still significant, decline was observed in adults aged 19–59 years (1109 studies, 111.4 million subjects; from 7.6% in 1973–1992 to 5.0% in 2006–2021, APC=−2.44, 95% CI −2.86 to –2.02). HBsAg seroprevalence increased in persons aged ≥60 years (212 studies, 5 48 220 subjects) according to APC analysis (APC=2.84, 95% CI 1.20 to 4.50), although the period estimates of HBsAg seroprevalence decreased from 7.0% in 1973–1992 to 4.6% in 2006–2021.

Both rural (657 studies, 4.3 million subjects) and urban (2323 studies, 46.7 million subjects) populations showed significant declines in HBsAg seroprevalence during the study period (online supplemental table S2), with this trend being stronger in urban (from 7.3% in 1973–1992 to 3.4% in 2006–2021, APC=−4.34, 95% CI −4.74 to –3.93) than rural (from 10.6% in 1973–1992 to 5.1% in 2006–2021, APC=−3.01, 95% CI −3.78 to –2.24) areas (online supplemental figure S6).

### Trends in HBsAg seroprevalence in high-risk groups

Five hundred and forty publications reported HBsAg seroprevalence for high-risk populations, of which 83 publications included both general and high-risk populations, but reported HBsAg prevalence independently. With respect to high-risk groups, 43, 66, 23, 21, 17, 64 and 328 studies for PLWH, IDU, sex workers, MSM, prisoners, migrant workers and hospital patients, respectively, were included. Decreasing trends of HBsAg seroprevalence were observed in all high-risk populations, both between the two main periods 1993–2005 and 2006–2021 (online supplemental table S3), as well as in APC analyses (online supplemental figure S7), with the notable exceptions of prisoners. Meta-analysis estimated HBsAg seroprevalence in 2006–2021 ranged from 15.0% and 10.7% in IDUs and PLWH, respectively, down to 4.8% in migrant workers.

### Meta-regression and sensitivity analyses

Determinants of HBsAg seroprevalence in China were evaluated in a meta-regression model (online supplemental tables S5–S7), confirming calendar period, age, sex and geographical area as significant independent determinants of HBsAg seroprevalence. Furthermore, HBsAg seroprevalence was confirmed to be significantly elevated in all high-risk groups versus the general population, most notably IDU (online supplemental table S7). In contrast, type of HBV assay (ELISA vs other), publication language (Chinese vs English), study grade and sample size were not significantly associated with HBsAg seroprevalence after accounting for geography and calendar period.

In two sensitivity analyses, the first focusing solely on ‘Grade A’ studies (including 22.5% of studies and 55.8% of subjects), and the second excluding blood donors and healthcare professionals (including 94.8% of studies and 95.9% of subjects), the main findings were materially unchanged (online supplemental tables S8 and S9, respectively). We observed interstudy heterogeneity across all strata studied in the general population (*I* 2>90%, p<0.0001) (table 1; online supplemental tables S1 and S2). We also identified publication bias in some, but not all, strata studied (online supplemental tables S3 and S4). Studies with smaller sample sizes tended to report a lower HBsAg prevalence.

### Estimated HBsAg seroprevalence in 2021

HBsAg seroprevalence in 2021 was estimated nationwide and according to significant determinants from the meta-regression model, namely geographical region, age and sex. From the overall linear regression model, nationwide HBsAg seroprevalence was 3.0% (95% CI 2.7 to 3.4%) and ranged across the six mainland regions from 1.4% (95% CI 0.7% to 2.2%) in North China to 3.4% (95% CI 2.6% to 4.2%) in South-West China (table 2). By weighing region-specific HBsAg prevalence in 2021 by regional population size, the nationwide HBsAg seroprevalence remained very consistent, namely 3.0% (95% CI 2.1% to 3.9%), equivalent to 43.3 million (95% uncertainty interval 30.7 to 55.9) HBV carriers in China in 2021 (table 2). The 2021 HBsAg seroprevalence estimate was (3.5%, 95% CI 2.9% to 4.0%) for men and (4.1%, 95% CI 3.7% to 4.5%) for women (table 2), and increased with age, being 0.3% (95% CI 0.2% to 0.4%), 1.0% (95% CI 0.8% to 1.1%), 4.7% (95% CI 4.3% to 5.1%) and 5.6% (95% CI 4.7% to 6.5%) for subjects aged under 5 years, 5–18 years, 19–59 years and ≥60 years, respectively. In 2021, the HBsAg prevalence was predicted as 4.8% (95% CI 4.2% to 5.1%) and 2.4% (95% CI 2.1% to 2.6%) for rural and urban areas, respectively.

**Table 2:** Estimates of HBsAg prevalence and number of HBV-infected people in 2021 in China, by region/territory, sex and age

Region/territory	Population size in 2021*	Estimated HBsAg prevalence in 2021 (%, 95% CI)	Estimated number of HBV-infected people in 2021 (million, 95% UI)
China mainland			
South Central China	409 783 486	3.2 (2.4 to 3.9)	12.9 (9.8 to 16.1)
East China	423 469 844	3.2 (2.5 to 3.9)	13.5 (10.4 to 16.7)
South-West China	205 148 550	3.4 (2.6 to 4.2)	6.9 (5.3 to 8.6)
North China	169 334 110	1.4 (0.7 to 2.2)	2.4 (1.1 to 3.8)
North-West China	103 527 786	2.6 (1.5 to 3.7)	2.6 (1.4 to 3.7)
North-East China	98 514 948	2.6 (1.4 to 3.8)	2.7 (1.5 to 3.9)
Taiwan	23 561 236	6.6 (2.7 to 10.4)	1.5 (0.6 to 2.5)
Hong Kong**†**	7474 200	8.4 (6.6 to 10.2)	0.6 (0.5 to 0.8)
Nationwide	1440 814 160	3.0 (2.1 to 3.9)	43.3 (30.7 to 55.9)
Sex			
Male	723 340 000	3.5 (2.9 to 4.0)	25.2 (21.1 to 29.2)
Female	688 440 000	4.1 (3.7 to 4.5)	28.2 (25.8 to 30.6)
Age (years)			
Under 5	63 124 810	0.3 (0.2 to 0.4)	0.2 (0.2 to 0.2)
5–18	245 679 125	1.0 (0.8 to 1.1)	2.3 (2.1 to 2.7)
19–60	834 004 694	4.7 (4.3 to 5.1)	39.6 (36.2 to 42.9)
≥60	177 594 440	5.6 (4.7 to 6.5)	9.9 (8.3 to 11.5)

*Population size data by region and sex were obtained from the seventh national census in 2020. Population size data by age were obtained from the sixth national census in 2010.

†The slope of linear regression model was not statistically significant.

HBsAg, HBV surface antigen; UI, uncertainty interval.

## Discussion

In this large meta-analysis covering more than 231 million people over the last four decades, we described the prevalence of chronic HBV infection in China and described wide variations in HBsAg prevalence by region, province, gender, age, rural/urban status and high-risk populations. We observed a significant and continuous decrease in HBsAg seroprevalence at the national level and for most subpopulations. However, there remained considerable variation in estimates of HBsAg seroprevalence across China in 2021, from below 1.5% in North China, to >3% in South-West China and >6% in Taiwan and Hong Kong. Children experienced the most pronounced decrease in prevalence, whereas a concurrent increase in HBsAg seroprevalence was observed in older persons (≥60 years).

In the last four decades, the country has spent considerable effort combatting HBV through immunisation.^18^ Briefly, China’s immunisation strategy began in 1984 with licensure of plasma-derived hepatitis B vaccine,^19^ followed by the licensure and national implementation of a recombinant vaccine in 1992. In 2002, China integrated HBV into its Expanded Program on Immunization (EPI), making the vaccine available at no cost to children aged <15 years,^20^ and a catch-up vaccination programme was launched in 2009. In 2011, China launched a programme to prevent mother-to-child transmission of HBV in 1156 counties.^21^ All pregnant women are tested systematically for HBV. For HBV-infected pregnant women, peripartum antiviral prophylaxis is administered after the twenty-eighth week of pregnancy.^22^ In addition, children born to these women receive free hepatitis B immunoglobulin within 12 hours postbirth followed by three doses of the vaccine.^18^ In 2015, the programme was rolled out nationally. This initiative also encompassed timely birth dose coverage of the HBV vaccine, which surged from 22.2% in 1992 to 95.6% in 2015.^18^ These efforts are expected to have driven the substantial decreases in HBV infection observed over time in China.

We estimated that nationwide HBsAg prevalence has decreased from around 10% before 1984 to 3.0% in 2021. Our historical nationwide estimates are comparable to that of the largest previous meta-analysis, based on 167 publications and 9.9 million subjects, that estimated a HBsAg seroprevalence of 5.5% in 2013 (our estimate for that year is ~5%),^14^ but tended to be slightly lower in comparison to a worldwide modelling study that suggested a 6.1% prevalence of HBV infection in mainland China in 2016^23^ (our estimate for that year is ~4%), as well as the largest previous national surveys (the data from which are nonetheless included in this meta-analysis, making direct comparisons not easy to interpret) that have been undertaken at certain time points.^14 23–25^ For example, national serosurveys conducted in 1992 and 2006 reported HBsAg prevalence of 9.8% (comparing with our 1992 estimate of ~8%), and 7.2% (comparing with our 2006 estimate of about 6%) in Chinese adults aged 1–59 years, respectively.^9^ These differences may be driven by the distribution of included participants in terms of geography, age and sex, all of which were confirmed by our meta-regression to be significant sources of heterogeneity in studies of HBsAg seroprevalence, even after accounting for strong temporal decreases. Of note, our national estimate for 2021 was similar even in a hierarchical model weighting region-specific HBsAg seroprevalence by region-specific populations, leading additional support to its representativeness of the Chinese population.

We observed a 7.7 annual per cent decrease per year in HBsAg seroprevalence in children aged <5 years during the past four decades, which is especially important given that seroprevalence of HBsAg among children under 5 years is considered by WHO as a surrogate indicator of the cumulative incidence of chronic HBV infection among adults.^26^ The estimate of HBsAg seroprevalence was 0.3% in this population in 2021, which was lower than the global average of 1.3% that was estimated by WHO in 2015 but still above the global goal of <0.1% by 2030.^27^ National surveys in 1992, 2006 and 2014 reported HBsAg seroprevalence of 9.9%, 1.0% and 0.3%, respectively, in children aged 1–4 years,^9^ which are consistent with our findings. In 2014, the national survey covering >30 000 people aged 1–29 years gave a HBsAg prevalence of 2.6%.^9^ Of note, we detected a significant increase in HBV prevalence among persons ≥60 years, although the period estimates of HBsAg seroprevalence from meta-analysis showed a decreasing trend. The older people have not been included in previous nationwide surveys.^7^ Positivity for HBsAg in older people seldom reflects recent acquisition of HBV infection, but rather long-lasting infection acquired in early childhood.^28^ Even in the most recent studies, people aged ≥60 years were all born before 1960, that is, 20–30 years before the initiation of HBV vaccination. In addition to the absence of preventive approaches at that time, the high background of HBV prevalence, due to larger family size (a Chinese baby boom occurred before 1960) and the resulting vertical (mother-to-child) and horizontal early childhood transmission, are expected to have contributed to elevate HBV seroprevalence in older birth cohorts. Indeed, an increase of HBV infection in older people is in line with our previous modelling study, where we found that the incidence of HBV-related liver cancer (mean age of approximately 60 years) increased significantly from 1990 to 2017 and is likely to increase further in the next decade.^29^ Increasing access to HBV diagnosis and the growing life expectancy might also contribute to this increase.^10^

In studies that reported stratification by sex, HBsAg seroprevalence was significantly higher in men than women. However, the rate of decline in HBsAg was twice as fast in men as in women during the study period (−3.90 vs −1.82), so that sex-specific differences reduced over time, and there were no remaining differences observable in 2021 estimates. There have been more reports of HBsAg seroprevalence in women than in men, driven by large serosurveys among pregnant women. Recently, a meta-analysis of 4 million women (compared with 121 million women included in our sex-specific analyses) across China estimated HBV seroprevalence at 7% in pregnant women during the period between 2016 and 2021,^30^ which is somewhat higher than our estimate that decreased from about 5% to 4% during this period. Our estimate for female HBsAg prevalence during this period was influenced by a single large report of 90 million pregnant women, that reported a persistent decrease in HBsAg seroprevalence (7.3% to 5.4%) between 2015 and 2020.^31^

The decrease in HBV infection rate was observed almost ubiquitously across all provinces in China, although with differing magnitude. However, we show that HBV seroprevalence remains highly heterogeneous across the country. In mainland China, we found that the overall HBsAg seroprevalence varied more than 3.5-fold at the province level, and the between-province disparity did not disappear in our most current estimates. The highest prevalence in the latest study period was observed in Tibet, followed by other provinces mostly clustered in southern China (Hainan, Jiangxi, Fujian, Guangxi, Guangdong and Qinghai), whereas the lowest seroprevalence was observed in Beijing and Shanxi. This large geographical heterogeneity is consistent with that observed in a very recently published study of pregnant women across 31 provinces of China.^31^ The disequilibrium in HBV infection rate across mainland China may have several explanations, predominantly the coverage and initiating time of HBV vaccination. In 1999, a national EPI review showed that the coverage of timely birth-dose (HepB_1_) and three-dose (HepB_3_) vaccine among 1-year-old children was 69.0% and 99.0% in Beijing, respectively, whereas the coverage rate for 2-year-old children was 7.8% and 2.1% in Tibet.^32^

HBV is still endemic in Taiwan and Hong Kong, in which the HBV infection rate was observed to be higher than mainland China. Both Taiwan and Hong Kong introduced HBV vaccination before 1990, and the protective effect of vaccine has been seen in the younger generations.^33 34^ The relatively high HBV prevalence in the two regions might be mainly due to high historical prevalence of HBV infection,^23 33 35^ despite an excellent vaccination coverage.^36^ Indeed, before the launch of the universal hepatitis B vaccination in 1984, the prevalence of HBsAg in the general population of Taiwan was 11%–20%, the highest known worldwide.^35^ In our study, we observed a significant reduction in HBV infection rate in Taiwan, whereas a non-significant decrease in Hong Kong. However, only a small number of studies (n=13) were included for Hong Kong, with few data prior to 1993. Of note, a very recently published study that has been included in our meta-analysis reported an approximately 50% reduction (10.0% to 5.3%) in HBsAg prevalence in Hong Kong between 2000 and 2019,^37^ suggesting that HBsAg, although still relatively high compared with other regions of China, is also decreasing in Hong Kong.

We estimated that there remain approximately 43.3 million chronically HBV-infected people in China in 2021. China therefore still faces challenges to achieving the global goal of hepatitis B elimination by 2030. Moreover, it is estimated that only 18.9% of HBV-infected cases are diagnosed and 12.6% are treated over their lifetime.^11^ The WHO-2030 mortality and programmatic targets will be difficult to achieve in China if the current screening and linkage-to-care strategy is not rapidly expanded.^38^ For the regions that still have relatively high HBsAg prevalence rates, more targeted strategies are warranted to reduce the disease burden of HBV infection. For example, the imperative for Tibet is that of continuously expanding the coverage of HBV vaccine. Indeed, only in early 2004 did Tibet begin offering universal HepB_1_ vaccination and the coverage has increased to >90% in infants as of 2016.^39^ With persistent investment and increasing HBV vaccination coverage, HBV prevalence in Tibet is likely to decline soon. For provinces in South Central China and East China such as Hainan, Guangdong, Guangxi, Jiangxi and Fujian, and Hong Kong and Taiwan, a universal HBV screening programme with high cost-effectiveness is needed,^38^ which is critical to identify more cases and is informative for subsequent allocation of medical sources for HBV-infection treatment.^40^ Early implementation of universal HBV screening would lead to more health benefits such as early diagnoses of HBV infection and HBV-related liver diseases. Moreover, universal screening is more important for the people who missed the birth dose of HBV vaccine before 2002.^41^

The decreasing trend in HBsAg seroprevalence was also observed among high-risk populations in China. This decrease is expected to be the consequence of infant vaccination and due to other initiatives such as improving blood safety, reduction in injecting and high-risk sex behaviours, education on HBV risk, and increase of HBV treatment.^42 43^ In contrast, we observed an increase in HBV infection rate among the prison population. Incarcerated individuals are exposed to a unique environment where various combinations of risk factors are ubiquitous, such as injection drug use, high-risk sexual activities, and sharing of sharp utensils and razors.^42^ Of note, in the recent study period, HBsAg prevalence is still higher in IDU and PLWH than in prisoners, indicating the two key populations at highest risk for HBV infection.

Of note, we observed a historical decrease in HBsAg prevalence among the general population already before the year of 1992. Indeed, in 1981, plasma-derived hepatitis B vaccine has been used in clinical practice to block the mother-to-children transmission.^44^ China then launched the national immunisation strategy in 1984 with licensure of plasma-derived hepatitis B vaccine.^19^ Moreover, the restriction on illegal blood trade, improvements in safety of blood donation and transfusion, and the increase in population awareness for hepatitis B in this period might also have contributed to this earliest decrease of HBV infection.

Some limitations of this study should be mentioned. First, like in many previously published meta-analyses of population prevalence,^45 46^ we pooled estimates despite significant between-study heterogeneity and publication bias. The use of a random-effects model, however, still allows the possibility of extrapolation outside the study population, and we addressed heterogeneity by meta-regression, and stratification and sensitivity analyses when possible. Second, we used HBsAg seroprevalence as the main marker indicating undergoing HBV infection in the present study, whereas other biomarkers such as anti-core antigen of HBV (HBc) or viral load were not considered, limiting the accuracy of the infection status in tested individuals, especially those with potentially occult infection. Third, because the studies included in this meta-analysis spanned nearly half a century, the sensitivity of HBsAg assays might have improved over time, resulting in an underestimation of the extent of the declines. Fourth, given the unavailability of individual-level data, groupings by age, sex, year and geography sometimes necessitated some approximation, and variables were not available for inclusion in meta-regression models with full mutual adjustment, nor to standardise HBsAg prevalence estimates by these variables over time.

In conclusion, thanks largely to investment of the Chinese government in widespread HBV vaccination, particularly in infants, China has experienced a remarkable decrease in HBV infection rate over the last four decades, especially visible in younger people. Although the prevalence in children under 5 years old has not yet reached the WHO impact target of ≤0.1%, China seems to be on track to reach the criteria for mother-to-child transmission elimination by 2030.^47^ HBsAg prevalence is however still high (≥5% in the most recent period) among high-risk populations and remains substantially heterogeneous nationwide. After initial excitement that HBV could be rapidly defeated, the reality is that China still has the heaviest burden of HBV chronic infection worldwide, that current diagnosis and treatment rates are far from the global 2030 targets, and that mortality rates from cirrhosis and hepatocellular carcinoma due to HBV are likely to remain high in the next decades, unless massive test-and-treat strategies are adopted to complement prevention measures.^23^ Ongoing efforts, involving strong public health commitment, medical investment, health education and individual awareness to HBV, especially in high-risk groups, are needed to further reduce the HBV morbidity and mortality burden, thereby approaching the global goal of HBV elimination.

## Data Availability

All data relevant to the study are included in the article or uploaded as supplementary information.
