# Supplementary material for: Changing prevalence of chronic hepatitis B virus infection in China between 1973 and 2021: a systematic literature review and meta-analysis of 3740 studies and 231 million people
Source: Gut. 2023 Oct 5;72(12):2354–63. doi: 10.1136/gutjnl-2023-330691 (PMC10715530; doi:10.1136/gutjnl-2023-330691)
Supplement: Supplementary data [file gutjnl-2023-330691supp001.pdf]

SUPPLEMENTARY MATERIALS

Changing prevalence of chronic hepatitis B virus infection in China between 1973 and 2021: a systematic literature review and meta-analysis of 3740 studies and 231 million people

Zhenqiu Liu, Chunqing Lin, Xianhua Mao, Chengnan Guo, Chen Suo, Dongliang Zhu, Wei Jiang, Yi Li, Jiahui Fan, Ci Song, Tiejun Zhang, Li Jin, Catherine de Martel, Gary M Clifford, Xingdong Chen

Search strategies

PubMed:

(("Hepatitis B, Chronic/epidemiology"[Mesh] OR "Hepatitis B/epidemiology"[Mesh] OR "Hepatitis D/epidemiology"[Mesh] OR "Hepatitis D, Chronic/epidemiology"[Mesh] OR "hepatitis b"[MeSH] OR "hepatitis b"[MeSH] OR "hepatitis b virus" OR "chronic hepatitis b" OR "hepatitis D"[MeSH Terms] OR "delta Hepatitis" OR "Superinfection Delta" OR "Delta Superinfection" OR "Hepatitis B Antigens "[MeSH] OR "hepatitis b surface antigens"[MeSH] OR "Australia Antigen" OR "hepatitis b core antigens"[MeSH] OR "Hepatitis B e Antigens "[MeSH] OR "hepatitis b surface antigen" OR "hepatitis b core antigen" OR "Hepatitis B e Antigen " OR "HBeAg" OR "HBsAg" OR "delta Antigen" OR "Hepatitis D Antigen" OR "HDAg" OR "Hepatitis D Virus " OR "Hepatitis Delta Virus"[MeSH] OR "Delta Virus" OR "Hepatitis Delta" OR "Delta Infection " OR "anti HBAg" OR "Labrea Disease" OR "Hepatitis B Antibodies"[MeSH] OR "Hepatitis B Antibody" OR "Hepatitis B Virus Antibodies" OR "HbsAg antibodies" OR "anti- HBc antibodies" OR "Anti-HBAg" OR "seromarker" OR "anti-HBe " OR "hepatitis antibodies" OR "anti-hepatitis b virus antibodies" OR "anti-hepatitis b virus antibody") AND ("prevalence"[MesH] OR "prevalence" OR "sero-prevalence" OR "seroepidemiologic studies"[MeSH] OR "sero survey*" OR "health survey*" OR "health surveys"[MeSH] OR "seroepidemiologic study"OR "Comparative Study" [Publication Type] OR "serosurveillance" OR "Epidemiology"[MeSH] OR "Hepatitis B, Chronic/statistics and numerical data"[Mesh] OR "Cohort Studies"[Mesh] OR "Longitudinal Studies"[Mesh] OR "Follow-Up Studies"[Mesh] OR "Prospective Studies"[Mesh] OR "Cross-Sectional Studies"[Mesh] OR "Surveys and Questionnaires"[Mesh] OR "Medical Records"[Mesh] OR "Registries"[Mesh] OR "Disease Notification"[Mesh] OR "Sentinel Surveillance"[Mesh]) NOT ("Carcinoma, Hepatocellular"[Mesh] OR "Liver Cirrhosis"[Mesh] OR "Case Reports"[Publication Type] OR "Editorial"[Publication Type] OR "Clinical Trial"[Publication Type] OR "Review"[Publication Type] OR "Meta-analysis"[Publication Type] OR "treatment" OR "therapeutics"[MeSH]) AND (Humans[Mesh]) AND (China OR Chinese))*

Web of Science:

(TI=chronic viral hepatitis B OR TS=chronic viral hepatitis B OR TI=Hepatitis B OR TS=Hepatitis B OR TI=Hepatitis B virus OR TS=Hepatitis B virus OR TI=Hepatitis B surface antigen OR TS=Hepatitis B surface antigen OR TI= Hepatitis Core Antigen OR TS= Hepatitis Core Antigen OR TI= Hepatitis Be Antigen OR TS= Hepatitis Be Antigen OR TI=HBsAg OR TS=HBsAg OR TI=HBcAg OR TS= HBcAg OR TI=HBeAg OR TS=HBeAg OR TI=hepatitis B co-infection OR TS=hepatitis B co-infection OR TI=Delta virus superinfection OR TS=Delta virus superinfection OR TI=Delta antigen OR TS=Delta antigen OR TI=Hepatitis D OR TS=Hepatitis D OR TI=Hepatitis D virus OR TS=Hepatitis D virus OR TI=Hepatitis D superinfection OR TS=Hepatitis D superinfection) AND (TI=prevalence OR TS=prevalence OR

TI=seroprevalence OR TS= seroprevalence OR TS=seroepidemiological study OR TI=seroepidemiological study OR TI=sero-survey OR TS=sero-survey* OR TI=serosurvey OR TS=serosurvey OR TI=sero survey* OR TS=sero survey* OR TI="Comparative Study" OR TI="serosurveillance" OR TI="Epidemiology" OR TI="Cohort Studies" OR TI="Longitudinal Studies" OR TI="Follow-Up Studies" OR TI="Prospective Studies" OR TI="Cross-Sectional Studies" OR TI="Surveys and Questionnaires" OR TI="Medical Records" OR TI="Registries" OR TI="Disease Notification" OR TI="Sentinel Surveillance" OR TS="Comparative Study" OR TS="serosurveillance" OR TS="Epidemiology" OR TS="Cohort Studies" OR TS="Longitudinal Studies" OR TS="Follow-Up Studies" OR TS="Prospective Studies" OR TS="Cross-Sectional Studies" OR TS="Surveys and Questionnaires" OR TS="Medical Records" OR TS="Registries" OR TS="Disease Notification" OR TS="Sentinel Surveillance") NOT (TS=Hepatitis C OR TI=Hepatitis C OR TI=Animals OR TS=Animals) AND (TS=China OR TS=Chinese OR TI=China OR TI=Chinese)*

EmBase:
(exp Hepatitis B virus/ or exp hepatitis B/ or exp hepatitis B surface antigen/ or Hepatitis B infection.mp. or Hepatitis B antibodies.mp. or exp hepatitis B antibody/ or Hepatitis B core antigen.mp. or exp hepatitis B core antigen/ or Hepatitis B e antigen.mp. / or chronic hepatitis B infection.mp. or Hepatitis D.mp. or hepatitis D virus.mp. or exp Hepatitis delta virus/ or hepatitis D co-infection.mp. or hepatitis D superinfection.mp. or exp delta agent hepatitis/ or hepatitis B antibody.mp. or Anti hepatitis b antibody*.mp. or delta superinfection.mp. or exp hepatitis delta antigen/) and (seroepidemiology/ or seroepidemiological studies.mp. or seroprevalence/ or prevalence.mp. or prevalence/ or seroprevalence.mp. or seroprevalence/ or exp health survey/ or sero survey*.mp. or serologicalsurvey*.mp. or disease prevalence.mp. or comparative studies.mp. or comparative study/ or sero survey.mp.) and China.mp. [mp=title, abstract, heading word, drug trade name, original title, device manufacturer, drug manufacturer, device trade name, keyword, floating subheading word, candidate term word]*

WanFang (Chinese database):
主题:(乙肝 OR 乙肝病毒 OR 乙型肝炎 OR 乙肝表面抗原) AND 主题:(感染率 OR 阳性率) AND 全部:(流行病学调查 OR 血清学调查 OR 人群调查 OR 调查)

CNKI (Chinese database):
SU=('乙肝'+'乙肝病毒'+'乙型肝炎'+'乙肝表面抗原')('感染率'+'阳性率') AND FT=(流行病学调查+血清学调查+人群调查+调查)*

Definitions used in this study

Term	Definition
**Population category**	
High-risk population	People living with HIV; injecting drug users; men who have sex with men; sex workers; migrant workers; prisoners; hospital in-patients and out-patients.
General population	People who were not explicitly declared as high-risk population in the publications, such as students, workers, pregnant women, military personnel, health care workers and blood donors.
**Age**	
Under 5	People who have been explicitly reported as aged 0-4 years; infants; and children in kindergartens.
5-18 years old	People who have been explicitly reported as aged 5-18 years; adolescents; students in primary schools, junior high schools, and senior high schools.
19-59 years old	Adults who have been explicitly reported as aged <60 years; workers; students in colleges; blood donor; pregnant women; health workers; military personnel; people taking premarital check-up.
≥60 years old	Adults who have been explicitly reported as ≥60 years; retiree.
**Rural/urban status**	
Rural area	People living in villages and towns, and counties; farmers; migrant workers
Urban area	People living in cities but not migrant workers

Figure S1. The flow chart for screening eligible publications.

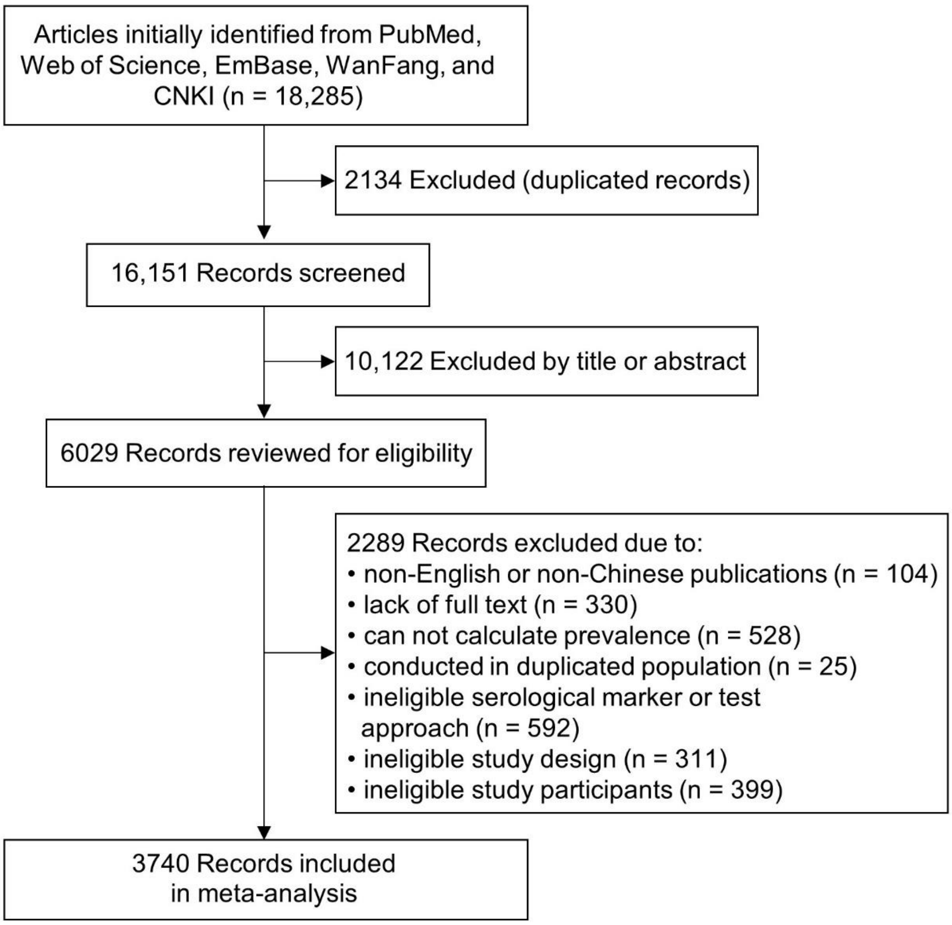

Ineligible study designs include meta-analysis, surveillance registration or national notifiable disease reports of incident hepatitis virus cases, and case studies. Ineligible study participants include those suffered from liver diseases, lived abroad, and were duplicated in more than one study. “Duplicated population” denotes the study subjects were overlapped or duplicated in different studies.

Figure S2. The number of studies included in this study by province.

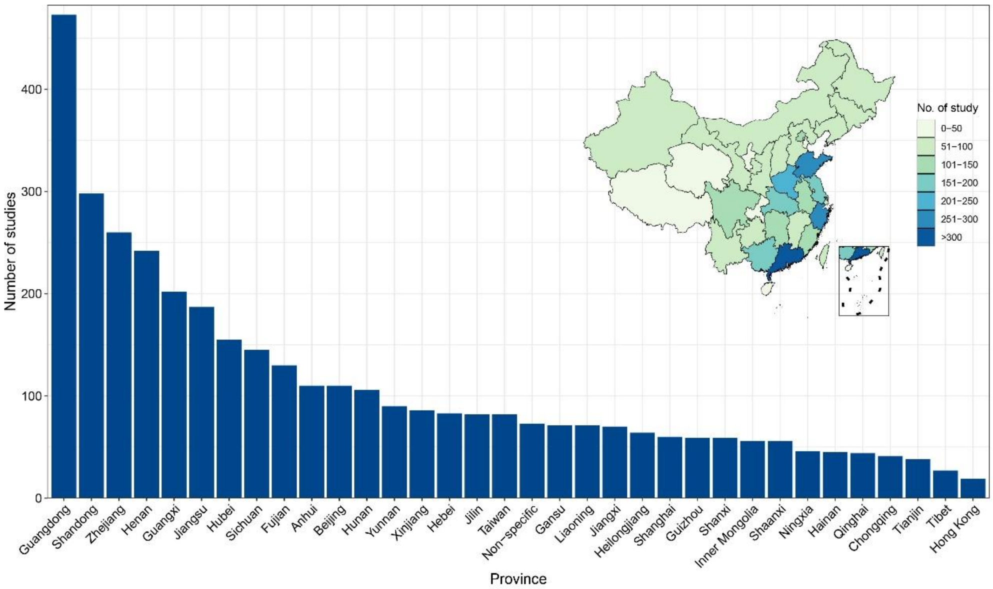

Non-specific means the province information was either unavailable or entangled (study participants were recruited from multiple provinces, e.g., *Song et al. PLoS One. 2014 Apr 8;9(4):e94528.*)

Figure S3. The number of included studies, by study year and populations.

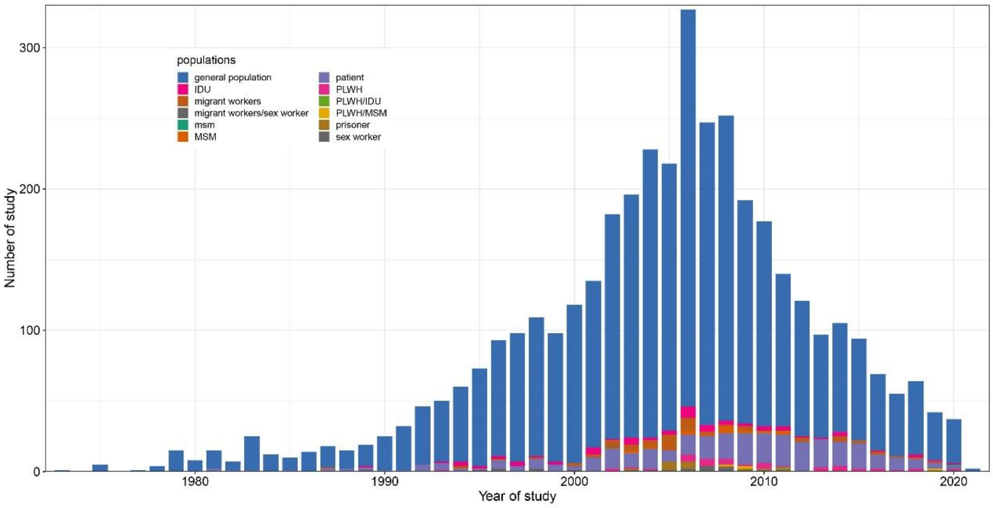

IDU, interjecting drug users; MSM, men who have sex with men; PLWH, people living with HIV. Patient included inpatients and outpatients whereas patients who were diagnosed with any liver diseases were excluded.

Figure S4. Trends in HBsAg seroprevalence, by mainland regions of China.

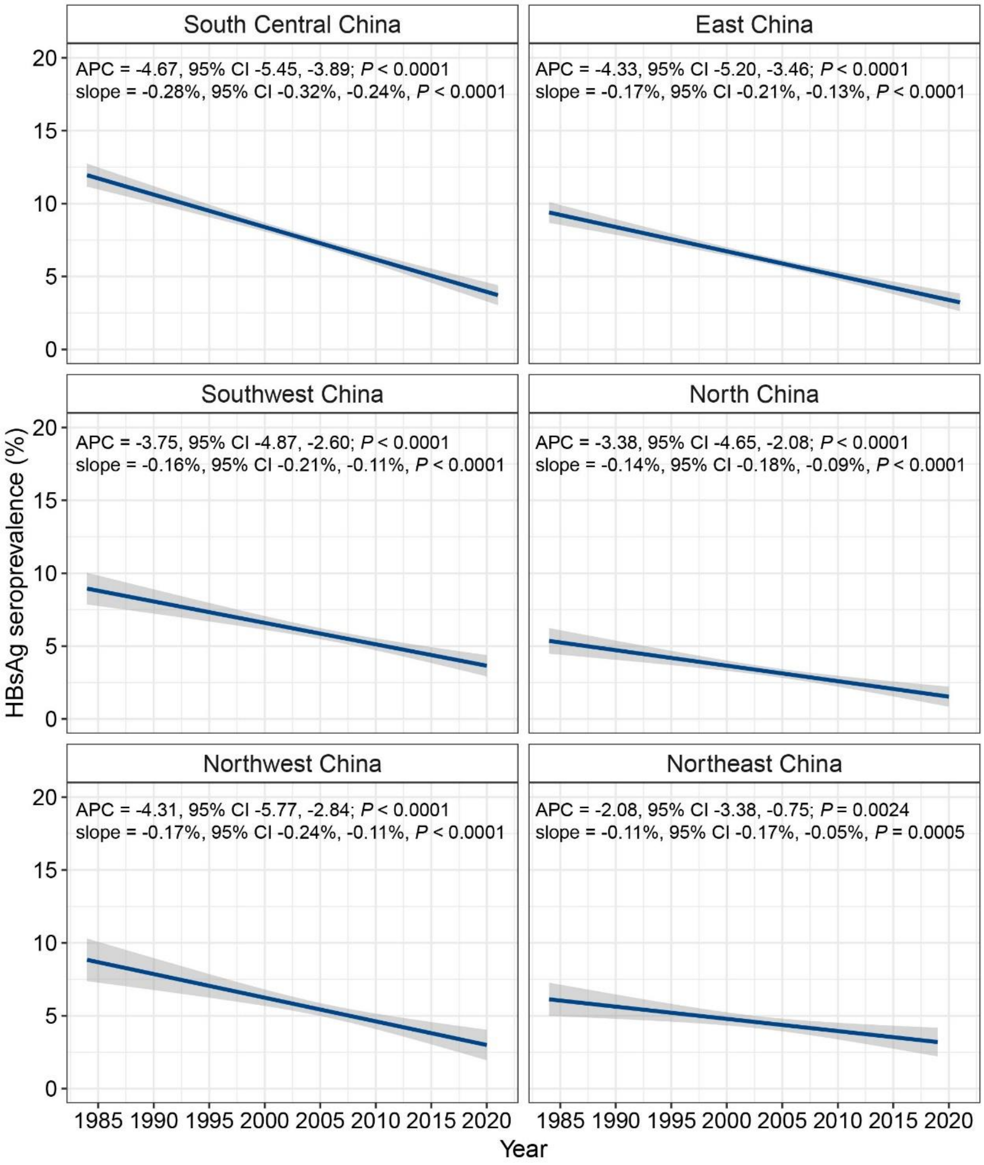

The estimates of HBsAg prevalence from included studies were fitted with the year of study using linear models. The blue lines were fitted lines and the gray shadows denoted the 95% confidence intervals of the fitted values.

Figure S5. Trends in HBsAg seroprevalence in China, by province/territory.

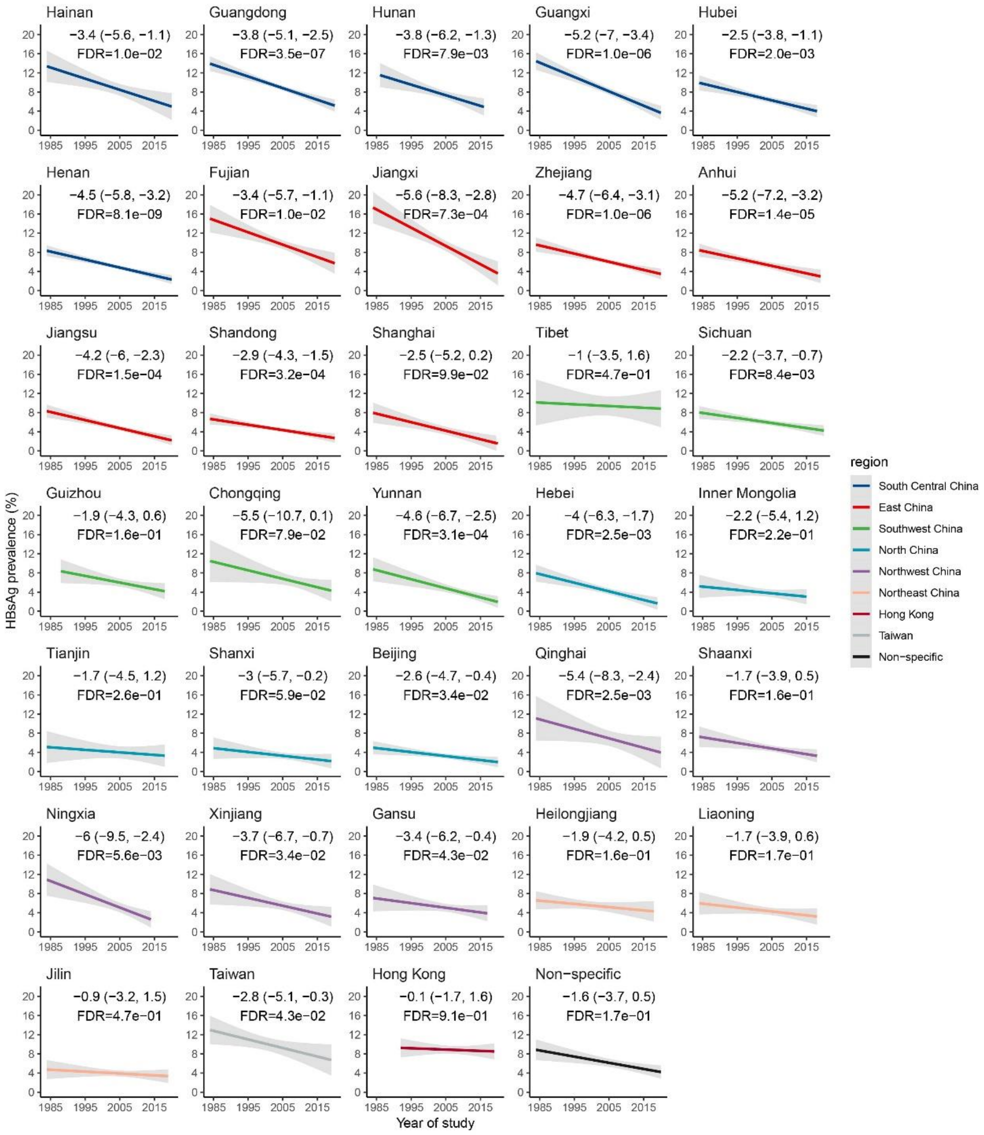

The estimates of HBsAg prevalence from included studies were fitted with the year of study using linear models. The gray shadows denoted the 95% confidence intervals of the fitted values. Numbers shown in the figure were APCs followed by its 95% confidence intervals and FDR values.

Figure S6. Trends in HBsAg seroprevalence in China, by rural/urban status.

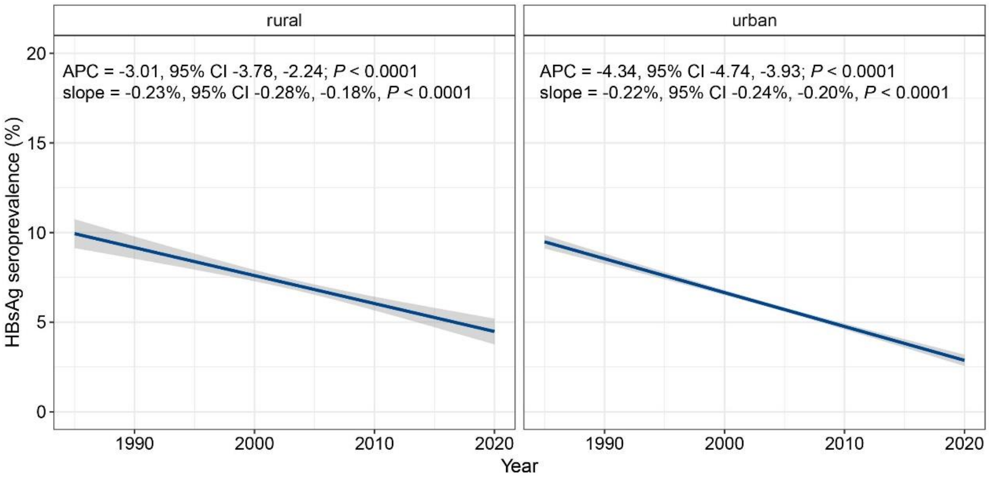

The estimates of HBsAg prevalence from included studies were fitted with the year of study using linear models. The blue lines were fitted lines and the gray shadows denoted the 95% confidence intervals of the fitted values.

Figure S7. Trends in HBsAg seroprevalence among high-risk populations in China.

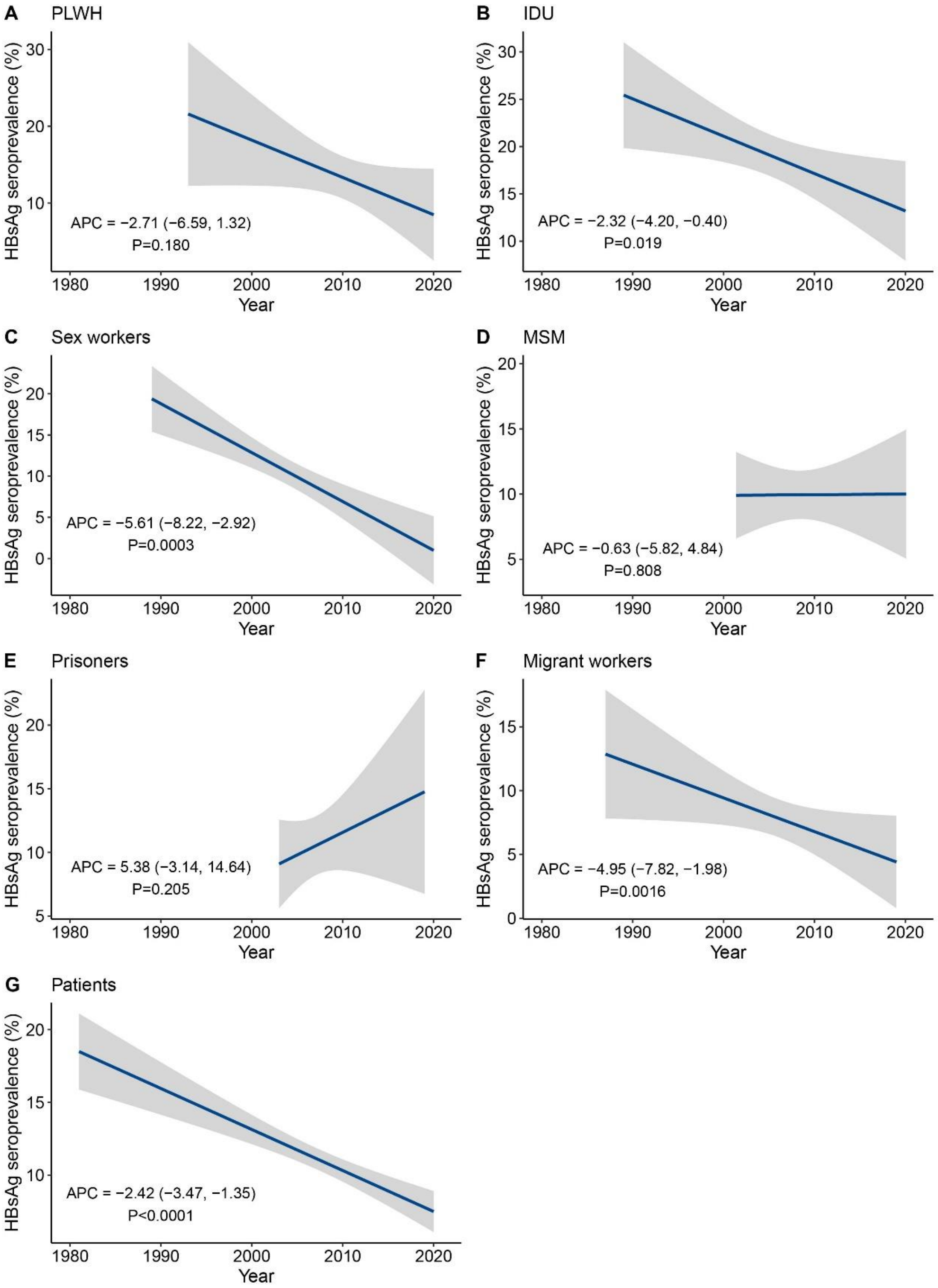

The estimates of HBsAg prevalence from included publications were fitted with year of study using linear models. The blue lines were fitted lines and the gray shadows denoted the 95% confidence intervals of the fitted values.

Table S1. The HBsAg seroprevalence among general population in China, by province and calendar period.

	**No. of study**	**Sample size**	**HBsAg seroprevalence (%, 95% CI) by calendar period***					
			**1973-1992**		**1993-2005**		**2006-2021**	
			Sample size	HBsAg seroprevalence (%, 95% CI)	Sample size	HBsAg seroprevalence (%, 95% CI)	Sample size	HBsAg seroprevalence (%, 95% CI)
**South Central China**								
Hainan	43	215,926	16,322	10.3 (7.5–14.0)	127,396	9.6 (7.4–12.3)	72,208	5.7 (4.2–7.8)
Guangdong	414	16,965,944	48,512	13.7 (9.3–19.5)	9,883,780	9.0 (8.1–9.9)	7,033,652	5.4 (4.6–6.3)
Hunan	90	1,053,138	7,371	19.9 (13.7–28.2)	361,393	7.4 (6.2–8.8)	684,374	4.9 (3.7–6.4)
Guangxi	183	3,136,653	32,120	13.0 (11.4–14.8)	1,355,839	8.3 (7.0–9.8)	1,748,694	4.0 (3.1–5.2)
Hubei	135	1,249,816	62,510	8.6 (7.0–10.5)	420,293	5.6 (4.6–7.0)	767,013	5.1 (4.5–5.9)
Henan	220	4,413,457	25,676	7.4 (5.6–9.6)	2,263,960	4.6 (4.1–5.2)	2,123,821	2.9 (2.4–3.5)
**East China**								
Fujian	115	1,140,360	21,576	15.1 (11.0–20.3)	321,359	9.4 (7.8–11.3)	797,425	6.2 (4.8–8.0)
Jiangxi	62	3,659,509	3,871	13.8 (11.3–16.7)	89,948	11.4 (9.7–13.3)	3,565,690	4.8 (3.4–6.7)
Zhejiang	235	7,173,665	17,343	8.0 (5.8–11.0)	1,249,604	6.0 (5.0–7.2)	5,906,718	3.2 (2.6–3.9)
Anhui	106	1,706,254	43,287	6.7 (5.4–8.3)	249,461	5.7 (4.9–6.7)	1,413,506	2.2 (1.3–3.5)
Jiangsu	164	3,854,354	12,358	6.2 (3.1–12.0)	405,953	4.1 (3.3–5.1)	3,436,043	3.0 (2.4–3.7)
Shandong	277	5,136,342	64,828	3.3 (1.4–7.6)	2,133,565	4.2 (3.8–4.6)	2,937,949	2.6 (2.1–3.1)
Shanghai	52	1,615,845	2,137	9.8 (8.6–11.2)	505,048	2.6 (1.7–3.9)	1,108,660	2.6 (1.9–3.6)
**Southwest China**								
Tibet	27	96,137	8,133	12.9 (8.7–18.8)	58,201	8.2 (5.3–12.5)	29,803	8.8 (6.4–12.1)
Sichuan	134	2,657,843	61,901	6.4 (4.5–9.0)	592,206	6.0 (5.3–6.9)	2,003,736	4.1 (3.3–5.0)
Guizhou	56	383,487	2,584	4.4 (2.3–8.4)	50,146	6.0 (4.4–8.2)	330,757	4.4 (3.8–5.2)
Chongqing	35	891,300	179	7.8 (4.7–12.8)	283,535	7.0 (5.0–9.9)	607,586	4.1 (2.7–6.1)
Yunnan	78	10,225,228	12,505	9.3 (4.9–17.1)	36,144	4.7 (3.2–6.9)	10,176,579	2.6 (2.1–3.1)
**North China**								
Hebei	82	2,002,940	11,418	8.3 (5.8–11.7)	345,754	3.4 (2.6–4.4)	1,645,768	2.6 (2.1–3.4)
Inner Mongolia	50	148,902	1,234	7.5 (2.2–22.3)	86,137	3.2 (2.4–4.2)	61,531	3.0 (2.2–4.1)
Tianjin	31	289,105	1,366	4.0 (2.4–6.7)	68,152	4.1 (3.0–5.5)	219,587	2.8 (2.0–3.9)
Shanxi	49	821,017	5,181	5.8 (5.2–6.5)	247,209	3.0 (2.3–3.8)	568,627	2.1 (1.6–2.7)
Beijing	95	5,013,798	7,142	4.0 (2.4–6.6)	241,628	2.5 (1.8–3.5)	4,765,028	2.3 (1.8–2.8)
**Northwest China**								
Qinghai	42	319,307	4,465	9.4 (7.9–11.1)	49,485	6.9 (4.9–9.8)	265,357	4.0 (2.8–5.7)
Shaanxi	49	742,601	460	9.6 (7.2–12.6)	503,895	4.6 (3.9–5.4)	238,246	3.7 (2.9–4.6)
Ningxia	42	413,484	673	7.7 (5.9–10.0)	151,828	5.5 (4.1–7.5)	260,983	3.0 (2.3–4.0)
Xinjiang	71	1,172,936	1,478	9.1 (6.6–12.5)	275,390	4.8 (3.8–6.0)	896,068	3.4 (2.5–4.6)
Gansu	67	615,218	1,983	12.7 (7.8–20.0)	214,442	4.7 (3.8–5.7)	398,793	3.4 (2.6–4.4)
**Northeast China**								
Heilongjiang	64	481,107	78,642	4.0 (2.6–6.1)	176,158	6.0 (4.8–7.5)	226,307	2.9 (1.9–4.3)
Liaoning	61	1,233,748	68,871	5.2 (3.1–8.8)	906,018	3.8 (3.1–4.6)	258,859	3.1 (2.4–4.1)
Jilin	78	1,046,479	11,300	4.9 (2.8–8.5)	579,193	3.1 (2.4–4.1)	455,986	3.2 (2.7–3.8)

* I^2 for interstudy heterogeneity >90% (P <0.0001) for all strata in this table.

Table S2. HBsAg seroprevalence among general population in China, by gender, age, rural/urban status, and calendar period.

	**No. of study**	**Sample size**	**HBsAg seroprevalence (%, 95% CI) by calendar period***					
			**1973-1992**		**1993-2005**		**2006-2021**	
			Sample size	HBsAg seroprevalence (%, 95% CI)	Sample size	HBsAg seroprevalence (%, 95% CI)	Sample size	HBsAg seroprevalence (%, 95% CI)
**Gender**								
Male	1,608	16,510,081	127,318	8.8 (8.0–9.6)	6,305,144	7.2 (6.9–7.5)	10,077,619	4.6 (4.4–4.8)
Female	1,802	120,723,325	134,682	6.3 (5.7–7.0)	6,700,796	5.0 (4.8–5.2)	113,887,847	3.9 (3.8–4.1)
**Age (years)**								
Under 5	409	996,331	31,105	5.0 (3.5–7.2)	460,328	1.7 (1.5–1.9)	504,898	0.6 (0.5–0.7)
5-18	910	5,145,475	69,277	10.1 (8.9–11.5)	3,127,019	5.3 (5.0–5.6)	1,949,179	2.4 (2.3–2.6)
19-59	1,109	111,438,412	111,930	7.6 (6.9–8.3)	4,257,712	6.4 (6.2–6.7)	107,068,770	5.0 (4.8–5.2)
60+	212	548,220	1,609	7.0 (5.8–8.3)	101,862	4.6 (4.0–5.4)	444,749	4.6 (4.1–5.1)
**Region**								
Rural	657	4,322,818	67,001	10.6 (9.3–12.2)	2,372,092	6.7 (6.2–7.2)	1,883,725	5.1 (4.7–5.5)
Urban	2,323	46,685,312	841,033	7.3 (6.6–8.0)	18,609,868	5.3 (5.1–5.5)	27,234,411	3.4 (3.2–3.5)

* I^2 for interstudy heterogeneity >90% (P <0.0001) for all strata in this table.

Table S3. HBsAg seroprevalence among high risk populations in China, 1973–2021.

**Population**	**No. of study**	**Sample size**	**HBsAg seroprevalence (%, 95% CI) by study period**							
			**1993–2005**				**2006–2021**			
			Sample size	HBsAg seroprevalence (%, 95% CI)	Egger-test P value#	I^2 (P value)	Sample size	HBsAg seroprevalence (%, 95% CI)	Egger-test P value#	I^2 (P value)
PLWH§	43	45,067	874	14.5 (6.6–28.9)	N.C.	90.3% (<0.0001)	44,193	10.7 (8.7–13.0)	**0.005**$	95.6% (<0.0001)
IDU†	66	50,797	21,815	20.4 (16.9–24.4)	**0.0002**$	95.7% (<0.0001)	28,982	15.0 (12.6–17.7)	0.120	95.4% (<0.0001)
Sex worker†	23	14,195	5445	13.4 (11.0–16.1)	0.309	79.5% (<0.0001)	8750	6.3 (4.9–8.2)	0.262	87.4% (<0.0001)
MSM§	21	13,005	681	12.2 (9.2–16.2)	N.C.	44.3% (0.146)	12,324	7.9 (6.2–9.9)	0.551	88.2% (<0.0001)
Prisoner§	17	28,627	14,467	8.1 (4.9–13.2)	N.C.	95.1% (<0.0001)	14,160	10.3 (7.8–13.5)	0.588	95.1% (<0.0001)
Migrant workers†	64	2,728,382	784,359	7.4 (5.7–9.6)	**0.028**$	99.8% (<0.0001)	1,944,023	4.8 (3.6–6.4)	0.345	99.9% (<0.0001)
Hospital patients†,*	328	3,678,205	361,399	11.8 (10.4–13.2)	**<0.0001**$	99.0% (<0.0001)	3,316,806	8.2 (7.5–9.0)	0.843	99.6% (<0.0001)

PLWH=people living with HIV; IDU=injecting drug user; MSM=men who have sex with men.
§No study was conducted before the year of 1993.
†Only one study was conducted in the period of 1973–1992, and therefore have been removed in this analysis.
*outpatients and inpatients, excluding patients with liver disease.
# P values were calculated from the Egger-test for publication bias and were adjusted by FDR method. The Egger-test was only performed for subgroups with more than 10 publications. N.C. denotes not calculable.
$ Studies with smaller sample sizes generally reported a lower HBsAg prevalence.

Table S4. Publication bias test for HBsAg seroprevalence in China by geography, sex, age, and region.

	Study period		
	**1973–1992**	**1993–2005**	**2006–2021**
	Egger-test P value*	Egger-test P value*	Egger-test P value*
**Geography**			
South Central China	0.853	0.853	**<0.0001**$
East China	0.664	**<0.0001**$	**<0.0001**$
Southwest China	0.579	0.156	0.059
North China	0.051	0.051	**0.030**$
Northwest China	0.579	0.067	0.351
Northeast China	0.362	0.296	0.296
Taiwan	0.811	0.352	0.067
Hong Kong	N.C.	0.362	0.664
**Nationwide**	0.952	**<0.0001**$	**<0.0001**$
**Gender**			
Male	**<0.0001**$	0.374	**0.005**$
Female	**<0.0001**$	**<0.0001**$	**<0.0001**$
**Age (years)**			
Under 5	0.896	0.339	0.199
5-18	0.754	0.108	0.102
19-59	**0.0002**$	**0.005**$	**<0.0001**$
60+	0.995	0.421	0.389
**Region**			
Rural	**0.0005**$	0.742	0.581
Urban	**0.0007**$	**<0.0001**$	**<0.0001**$

*P values were calculated from the Egger-test for publication bias and were adjusted by FDR method. The Egger-test was only performed for subgroups with more than 10 publications. N.C. denotes not calculable.

$ Studies with smaller sample sizes generally reported a lower HBsAg prevalence.

Table S5. Results of meta-regression for studies reporting HBV infection rate by age.

	Prevalence ratio (95% CI)	P value
**Age (years)**		
Under 5	Ref.	
5-18	1.40 (1.14–1.65)	0.017
19-59	2.08 (1.85–2.57)	<0.0001
≥60	2.84 (2.58–3.07)	<0.0001

In this analysis, 87.7% (n=2879) of total studies were excluded due to the unavailability of age.

Table S6. Results of meta-regression for studies reporting HBV infection rate by sex.

	Prevalence ratio (95% CI)	P value
**Sex**		
Male	Ref.	
Female	0.85 (0.80-0.90)	<0.0001

In this analysis, 50.1% (n=1645) of total studies were excluded due to the unavailability of sex.

Table S7. Results of meta-regression to assess the heterogeneity from other variables.

Characteristic	Prevalence ratio (95% CI)	P value
**Study period**		
1973-1992	Ref.	
1993-2005	0.78 (0.70–0.83)	<0.0001
2006-2021	0.51 (0.47–0.55)	<0.0001
**Sample size**		
<500	Ref.	
500-1000	1.01 (0.92–1.09)	0.872
1001-10000	1.07 (0.95-1.12)	0.335
>10000	1.02 (0.96-1.06)	0.744
**Region**		
South Central China	Ref.	
Taiwan	1.11 (0.92–1.26)	0.134
Hong Kong	1.09 (0.84–1.62)	0.428
Southwest China	0.83 (0.74–0.92)	0.007
Northwest China	0.78 (0.71–0.86)	<0.0001
East China	0.91 (0.83–0.95)	0.002
Northeast China	0.51 (0.46–0.55)	<0.0001
North China	0.50 (0.46–0.54)	<0.0001
**Populations**		
General population	Ref.	
IDU	4.22 (3.91–4.68)	<0.0001
PLWH	2.75 (2.37–3.12)	<0.0001
MSM	1.54 (1.28–1.94)	0.004
Patients	2.01 (1.61–2.41)	<0.0001
Sex workers	2.42 (2.11–2.74)	<0.0001
Prisoners	1.55 (1.21–1.89)	0.006
Migrant workers	1.41 (1.31–1.52)	<0.0001
**HBV assay**		
ELISA	Ref.	
Others	1.03 (0.92-1.12)	0.844
**Publication language**		
English	Ref.	
Chinese	1.08 (0.98-1.15)	0.102
**Study grade**		
A	Ref.	
B	1.03 (0.92–1.11)	0.483
C	1.08 (0.92–1.23)	0.105

tau^2 (estimated amount of residual heterogeneity): 0.6892
PLWH: people living with HIV; IDU: injecting drug user; MSM: men have sex with men. Ref., reference group. SE, standard error. ELISA, enzyme-linked immunosorbent assay

Table S8. HBsAg seroprevalence among general population in China, 1973-2021, based on the "Grade A" publications in each province (Sensitivity analysis 1).

**Region**	**Study period**					
	**1973–1992**		**1993–2005**		**2006–2021**	
	Sample size	HBsAg seroprevalence (%, 95% CI)	Sample size	HBsAg seroprevalence (%, 95% CI)	Sample size	HBsAg seroprevalence (%, 95% CI)
South Central China	40,478	11.2 (7.2-15.2)	6,002,198	6.2 (5.4-7.2)	5,218,121	3.6 (3.0-4.3)
East China	18,852	7.7 (4.8-12.0)	1,516,468	4.0 (3.3-4.8)	6,981,455	2.9 (2.4-3.6)
Southwest China	20,557	7.4 (5.8-9.4)	489,087	4.6 (3.5-5.9)	3,180,112	3.4 (2.6-4.4)
North China	5332	5.7 (3.8-8.3)	241,663	1.9 (1.2-2.9)	2,205,803	1.8 (1.4-2.2)
Northwest China	3622	8.6 (7.7-9.6)	155,854	5.9 (4.9-7.1)	997,541	2.9 (2.2-3.7)
Northeast China	15,140	3.2 (2.7-3.9)	307,148	3.6 (3.5-5.2)	242,782	2.7 (2.0-3.6)
**Nationwide**	165,953	8.0 (6.2-10.4)	8,832,107	5.3 (4.6-5.7)	116,067,748	3.1 (2.8-3.4)

Table S9. HBsAg seroprevalence among general population in China, 1973-2021, with the exclusion of blood donors and health care workers (Sensitivity analysis 2).

	**Study period**					
	1973-1992		1993-2005		2006-2021	
	Sample Size	HBsAg seroprevalence (%, 95% CI)	Sample Size	HBsAg seroprevalence (%, 95% CI)	Sample Size	HBsAg seroprevalence (%, 95% CI)
**South Central China**	184,370	11.3 (9.8-13.0)	11,388,361	7.4 (6.9-7.9)	10,932,416	4.7 (4.2-5.1)
Hainan	15,135	12.4 (11.9-12.9)	127,396	9.6 (7.4-12.3)	72,208	5.7 (4.2-7.8)
Guangdong	48,007	13.4 (8.9-19.7)	7,296,656	9.1 (8.2-10.0)	6,283,567	5.7 (4.9-6.7)
Hunan	7,371	19.9 (13.7-28.2)	331,225	7.2 (6.0-8.7)	584,930	5.2 (4.0-6.8)
Guangxi	27,444	13.6 (12.0-15.5)	1,168,473	8.4 (7.1-10.0)	1,672,352	4.1 (3.1-5.3)
Hubei	61,397	8.8 (7.0-11.0)	419,169	5.7 (4.6-7.1)	766,002	5.2 (4.5-6.0)
Henan	25,016	7.1 (5.3-9.4)	2,045,442	4.7 (4.2-5.2)	1,553,357	2.9 (2.3-3.6)
**East China**	156,450	7.9 (6.6-9.4)	4,838,252	5.2 (4.8-5.6)	16,925,032	3.4 (3.1-3.8)
Fujian	21,576	15.1 (11.0-20.3)	320,018	9.3 (7.7-11.2)	797,425	6.2 (4.8-8.0)
Jiangxi	3,871	13.8 (11.3-16.7)	89,948	11.4 (9.7-13.2)	3,501,290	5.0 (3.5-6.9)
Zhejiang	16,842	7.9 (5.5-11.1)	1,233,188	5.9 (4.9-7.2)	5,786,272	3.2 (2.6-4.0)
Anhui	37,309	6.5 (5.0-8.4)	249,461	5.7 (4.9-6.7)	840,821	2.4 (1.5-3.9)
Jiangsu	9,887	8.0 (6.0-10.8)	404,759	4.2 (3.4-5.2)	2,585,185	3.3 (2.7-4.0)
Shandong	64,828	3.3 (1.4-7.6)	2,035,830	4.1 (3.6-4.5)	2,305,379	2.7 (2.2-3.4)
Shanghai	2,137	9.8 (8.6-11.2)	505,048	2.7 (1.8-4.0)	1,108,660	2.6 (1.9-3.6)
**Southwest China**	83,230	7.7 (5.8-10.2)	1,015,621	6.0 (5.3-6.8)	12,950,625	3.8 (3.4-4.3)
Tibet	8,133	12.9 (8.7-18.8)	58,201	8.2 (5.3-12.5)	29,803	8.8 (6.3-12.1)
Sichuan	60,008	6.5 (4.4-9.4)	587,595	5.9 (5.1-6.8)	1,864,565	4.4 (3.6-5.4)
Guizhou	2,584	4.4 (2.3-8.4)	50,146	6.0 (4.4-8.2)	330,757	4.4 (3.8-5.2)
Chongqing	0	NA	283,535	7.0 (4.9-9.8)	548,921	3.9 (2.4-6.2)
Yunnan	12,505	9.3 (4.9-17)	36,144	4.7 (3.2-6.9)	10,176,579	2.5 (2.1-3.1)
**North China**	24,988	6.6 (5.1-8.5)	948,259	3.1 (2.7-3.5)	5,209,324	2.6 (2.3-2.9)
Hebei	11,418	8.3 (5.8-11.7)	311,069	3.4 (2.6-4.6)	1,159,765	2.9 (2.3-3.6)
Inner Mongolia	1,234	7.5 (2.2-22.3)	86,137	3.2 (2.4-4.2)	61,531	3.0 (2.1-4.1)
Tianjin	1,366	4.0 (2.4-6.6)	67,477	4.0 (2.8-5.5)	187,032	3.2 (2.4-4.4)
Shanxi	5,181	5.8 (5.2-6.5)	242,711	2.9 (2.2-3.8)	178,831	2.2 (1.7-2.8)
Beijing	5,789	5.2 (3.7-7.1)	240,865	2.5 (1.7-3.5)	3,622,165	2.4 (1.9-2.9)
**Northwest China**	9,059	10.0 (8.1-12.3)	1,181,587	5.1 (4.5-5.7)	2,059,447	3.5 (3.0-4.0)
Qinghai	4,465	9.4 (7.9-11.1)	43,492	7.1 (4.9-10.3)	265,357	4.0 (2.8-5.6)
Shaanxi	460	9.6 (7.2-12.6)	500,509	4.7 (4.0-5.5)	238,246	3.7 (2.9-4.6)
Ningxia	673	7.7 (5.9-10.0)	148,037	5.7 (4.0-7.9)	260,983	3.0 (2.3-4.0)
Xinjiang	1,478	9.1 (6.6-12.4)	275,107	4.7 (3.7-6.0)	896,068	3.4 (2.5-4.6)
Gansu	1,983	12.7 (7.8-20.0)	214,442	4.7 (3.8-5.7)	398,793	3.4 (2.5-4.4)
**Northeast China**	154,391	4.5 (3.1-6.4)	1,658,848	4.2 (3.6-4.9)	925,630	3.3 (2.9-3.47)
Heilongjiang	77,741	4.1 (2.4-6.7)	176,158	6.0 (4.8-7.4)	225,207	3.2 (2.2-4.6)
Liaoning	66,202	6.0 (3.4-10.3)	904,575	3.8 (3.0-4.6)	257,626	3.3 (2.5-4.2)
Jilin	10,448	4.0 (1.7-9.0)	578,115	3.1 (2.3-4.1)	442,797	3.3 (2.8-3.9)
**Taiwan**	26,957	12.2 (9.8-15.2)	497,590	10.8 (8.7-13.4)	264,606	5.0 (3.0-8.2)
**Hong Kong**	3,569	3.6 (3.0-4.2)	226,326	8.8 (7.4-10.4)	169,250	8.8 (7.9-9.7)
**Nationwide**	718,002	8.6 (7.9-9.4)	26,259,641	5.7 (5.5-6.0)	187,856,659	3.8 (3.6-3.9)
